# Medical Students Immersed in a Hyper-Realistic Surgical Training Environment Leads to Improved Measures of Emotional Resiliency by Both Hardiness and Emotional Intelligence Evaluation

**DOI:** 10.3389/fpsyg.2020.569035

**Published:** 2020-11-20

**Authors:** Allana White, Isain Zapata, Alissa Lenz, Rebecca Ryznar, Natalie Nevins, Tuan N. Hoang, Reginald Franciose, Marian Safaoui, David Clegg, Anthony J. LaPorta

**Affiliations:** ^1^Department of Cardiothoracic Surgery, University of Colorado, Anschutz Medical Campus, Aurora, CO, United States; ^2^Department of Biomedical Sciences, Rocky Vista University College of Osteopathic Medicine, Parker, CO, United States; ^3^Department of Military Medicine, Rocky Vista University College of Osteopathic Medicine, Parker, CO, United States; ^4^Western University of Health Sciences, College of Osteopathic Medicine of the Pacific, Pomona, CA, United States; ^5^Naval Readiness Training Command, Naval Medical Forces, Pacific, Twentynine Palms, CA, United States; ^6^Department of Surgery, Vail Valley Medical Center, Vail, CO, United States; ^7^Michael Tang Regional Center for Clinical Simulation, Touro University Nevada, Henderson, NV, United States

**Keywords:** Hardiness, Emotional Intelligence, resilience (psychological), hyper-realistic, medical student, military

## Abstract

**Background:**

Burnout is being experienced by medical students, residents, and practicing physicians at significant rates. Higher levels of Hardiness and Emotional Intelligence may protect individuals against burnout symptoms. Previous studies have shown both Hardiness and Emotional IntelIigence protect against detrimental effects of stress and can be adapted through training; however, there is limited research on how training programs affect both simultaneously. Therefore, the objective of this study was to define the association of Hardiness and Emotional Intelligence and their potential improvement through hyper realistic immersion simulation training in military medical students.

**Methods:**

Participants in this study consisted of 68 second year medical students representing five medical schools who were concurrently enrolled in the United States military scholarship program. During a six day hyper-realistic surgical simulation training course, students rotated through different roles of a medical team and responded to several mass-casualty scenarios. Hardiness and Emotional Intelligence were assessed using the Hardiness Resilience Gauge (HRG) and the Emotional Quotient Inventory (EQ-I 2.0) respectively, at two time points: on arrival (pre-event) and after completion of the course (post-event).

**Results:**

Hardiness and Emotional Intelligence scores and sub scores consistently improved from pre-event to post-event assessments. No difference in training benefit was observed between genders but differences were observed by age where age was more often associated with Emotional Intelligence. In addition, factor analysis indicated that the HRG and EQ-I 2.0 assessment tools measured predominately different traits although they share some commonalities in some components.

**Conclusion:**

This study indicates that Hardiness and Emotional Intelligence scores can be improved through immersion training in military medical students. Results from this study support the use of training course interventions and prompt the need for long term evaluation of improvement strategies on mitigating burnout symptoms.

## Introduction

Medical professionals, are expected to perform at an extraordinary level while working in a stressful environment where failure can be costly. Their continuous exposure to stressful environments increases their chances to develop symptoms of burnout (Adler et al., 2017; West et al., 2018). Burnout is a psychological syndrome comprised of feelings of exhaustion, depersonalization, and reduced professional efficacy that are detrimental to their professional performance (Maslach and Jackson, 1981). Symptoms of burnout often begin in medical school (Frajerman et al., 2019), continue in residency (Legassie et al., 2008; Garcia-Rodriguez et al., 2017) and are well established in practicing physicians (Shanafelt et al., 2012; West et al., 2018). Burnout affects upwards of 45% of this population. There are many individual characteristics that may contribute to an increased likelihood of developing burnout, including personality characteristics, and work attitudes (Maslach and Jackson, 1981). Individuals that have low levels of Hardiness, low Emotional Intelligence, poor self-esteem, and have avoidant coping mechanisms may experience higher rates of burnout (Dahlin et al., 2007; Garrosa et al., 2010; Galaiya et al., 2020).

Hardiness refers to one’s resilience and ability to cope with stressful and unexpected situations. Studies have shown that higher levels of resilience correlates to improved stress tolerance (Bartone, 1999; Kalantar et al., 2013; Sandvik et al., 2013) and may be protective effects against post-traumatic stress disorder (Bartone, 1999; Escolas et al., 2013) and burnout symptoms (Buck et al., 2019). Hardiness is characterized by challenge, the ability to be flexible and grow from failure, control, acknowledging that managing one’s life is always possible and commitment, having a sense of meaning and purpose with the ability to stay motivated (Kobasa, 1979).

Emotional Intelligence is also a set of modifiable skills that reflect individuals’ inherent differences rather than the trajectory of basic processes used for emotion regulation, therefore persons with higher Emotional Intelligence scores are better in regulating their emotions than people with lower scores (Peña-Sarrionandia et al., 2015). Increased Emotional Intelligence is a protective benefit against burnout (Szczygieł et al., 2012; Sarrionandia et al., 2018). Emotional Intelligence allows individuals to perceive, manage, express, and react to the emotions of oneself and those of others (Mayer et al., 1999). Although there are several competing models of Emotional Intelligence (Roberts et al., 2010), one that has received high public visibility is the one proposed by Bar-On in 1997 (Bar-On, 1997). The Bar-On model, has provided a theoretical framework for the development of the “Emotional Quotient Inventory” (EQI) assessment instrument which uses a self-report survey that evaluates the subjective perception of the participants. The EQ-I instrument evaluates five main components: intrapersonal, interpersonal, stress management, adaptability, and general mood that are further broken down into 15 subscale scores (Bar-On, 1997).

Higher levels of individual Hardiness and Emotional Intelligence may contribute to mitigating perceived stress and symptoms of burnout. Studies have shown both Emotional Intelligence (Gorgas et al., 2015; Shahid et al., 2018) and Hardiness (Maddi, 2002; Bartone et al., 2008; Kalantar et al., 2013) can be improved through training. However, there is no published research on how training can improve both facets at the same time. As noted, burnout can start as early as the medical student level, which is concerning as this population has only begun their journey. Therefore, it is crucial to assess if implementing Hardiness and Emotional Intelligence training at this stage could protect against burnout in residency and practice. Given the limited time health care professionals have for additional training, modifying both Hardiness and Emotional Intelligence simultaneously to reduce burnout at the earliest stage possible in their career, would be ideal.

Simulation training enhances knowledge, skills and appropriate coping behaviors of medical professionals along with improving patient related outcomes (Cook et al., 2011). Prior studies about stress management skills have shown limited effectiveness because the main goal has been the implementation of stress coping mechanisms rather than utilizing simulated stressful scenarios to prepare participants for future events (Ignacio et al., 2016). Based on these findings, and the success of the US Military in the use of simulation training for aviation and nuclear power management training purposes (Saunders et al., 1996), a similar approach was developed to help train future doctors. This training approach utilizes the same “stress inoculation” concept developed for the previous applications by recreating a high fidelity scenario that incorporates the whole magnitude of sensory stimuli within the context of the real event (LaPorta et al., 2017; Pierce et al., 2018; Hoang et al., 2020). These training interventions have been exclusive to military use; but recently applied for civilian use. Hyperrealistic stress inoculation training utilized in medical school education, can simulate stressful emergency medical scenarios in order to condition future doctors for such events throughout their medical careers.

The present study evaluates the short term effects of hyper realistic training and its impact on Hardiness and Emotional Hardiness, with the ultimate long term goal of improving the performance and wellbeing of medical personel throughout the duration of their careers. The objective of this study is to define the association of Hardiness and Emotional Intelligence and their potential improvement through the use of hyper realistic immersion simulation training in military medical students. The type of training presented in this study which was developed for military training purposes is expected to be transferred to the civilian domain in the near future, our study pioneers the objective evaluation of its effectiveness and repercussions which are the overall focus of this research effort.

## Materials and Methods

### Experimental Design

This project was designed as a prospective study to evaluate the conjoined effect of Hardiness and Emotional Intelligence after a hyper realistic surgical simulation training session. The hyper realistic surgical training session simulates the immersive environment of a mass-casualty event in a non-threatening military setting where the entire timeline from the simulated attack, situation assessment, triage, medical management is recreated in real time. The training event spans for six successive days where each day a different event scenario is recreated and individual roles are reassigned. This training session and study was conducted at Strategic Operations (STOPS), San Diego, CA. Strategic Operations is a current US Military provider of hyper realistic environments for surgical and combat training. Casualties are simulated using “Cut Suits” which provide a life-like patient provider interaction that enable surgical teams to practice realistically simulated life threatening injuries. Participants are not briefed in advance about the scenario, type of simulated casualties and number they will encounter. More information about the training program can be obtained in prior publications (LaPorta et al., 2017; Hoang et al., 2020). For our study, a pre-event (arrival) and post-event (after the last session on the 6th day) assessment of Hardiness and Emotional Intelligence was implemented to evaluate the effect of the training session. Hardiness was evaluated using the HRG assessment instrument by Multi-Health Systems Inc. (MHS) Toronto, Canada. Emotional Intelligence was evaluated using the EQ-I 2.0 assessment instrument provided by MHS as well.

### Participants

A total of 68 second year medical students enrolled at five Osteopathic Medical Colleges as members of the Health Professions Scholarship Program of the US Military participated in the study. These military medical students were enrolled at either of five Medical Colleges: A.T. Still University (ATSU), Kansas City University of medicine and Biosciences (KCUMB), Rocky Vista University (RVU), Touro university (TU), and Western University of Health Sciences (WUHS). All participants consented in writing and remained in the study. The study was approved by the Rocky Vista University Institutional Review Board (RVU IRB 2014-0001). All responses were matched across time points for each participant and were de-identified prior to analysis. Participants had access only to their individual scores and to aggregated average data from their cohort and all particiapants had the option to stop their participation in the intervention and the study. The study was performed in two sessions, one in 2018 and the second in 2019. Detailed participant demographics are presented in Table 1.

**TABLE 1:** Study participant demographics.

**Variable**	**Mean**	**Std. Dev.**	**Minimum**	**Maximum**
Age (years)	27.04	3.36	21	41

	**Frequency**	**Percent**	**Cumulative frequency**	**Cumulative percent**

**Sex**				
Female	17	25	17	25
Male	51	75	68	100
**Year**				
2018	35	51.47	35	51.47
2019	33	48.53	68	100
**University**				
ATSU	1	1.47	1	1.47
KCUMB	10	14.71	11	16.18
RVU	44	64.71	55	80.88
TU	4	5.88	59	86.76
WUHS	9	13.24	68	100

### Statistical Analysis

Descriptive statistics were estimated using SAS/STAT v.9.4 (SAS Institute Inc., Cary, NC, United states). Differences between pre-event and post-event Hardiness (HRG) and Emotional Intelligence (EQ-I2.0) were calculated for each sub score and the total per individual, standardized scores and sub scores were used exclusively.

Hardiness and Emotional Intelligence score variation patterns were explored by factor analysis. Factor analysis was performed though the Principal Components Factor Method. Two separate Factor analysis were performed: first, for the pre-event (t1) and post-event (t2) values, this included total and sub scores; second, for the difference values which included total and sub scores as well.

The additive effects of the pre-event and post-event (Time point) Hardiness and Emotional Intelligence scores along with age and sex were evaluated by Generalized Linear Mixed Models using PROC GLIMMIX. Each score was evaluated independently. The interaction effect of Sex by Time point was included. The session “year” was included in the model as a Random variable and the individual was defined as the experimental unit. For this first iteration, the model can be defined as:

(1) $$Standardizedscore\left(HRG,EQ-I2.0\right){}_{i\,j\,k\,l}$$

 =Age+iSex|jTimepoint+kyear+lεi⁢j⁢k⁢l

A second model iteration was performed on the differences where only the additive effects of sex and age were included (the time point effect is implied in the Δ and thus not included). This second iteration included the same Random effect of session “year” and the defined the individual as the experimental unit For this seconf ithereation, the model can be defined as:

(2) $$\Delta Standardizedscore\left(\Delta HRG,\Delta EQ-I2.0\right){}_{i\,j\,k}$$

 =Age+iSex+jyear+kεi⁢j⁢k

All errors were assumed as independent and normally distributed. Significance was declared at *P* ≤ 0.05. In addition, family wise significance was noted with a Bonferroni adjusted confidence threshold (0.05 divided by the number of tests performed per family of tests).

## Results

Pre-event, post-event and difference (Δ) scores and sub scores for Hardiness (assessed through the HRG instrument) and Emotional Intelligence (assessed through the EQ-I2.0 instrument) are displayed in Table 2. Pre-event scores and sub scores all consistently lower than the Post-event scores and sub scores. Standard deviation across the two instruments is very similar, this was expected since they are both standardized values. A similar pattern can be observed in the Δ scores and sub scores where all values are positive indicating a positive gain. Direct correlation of Hardiness vs EQ scores were all significant (*P* < 0.0001; pre-event *r* = 0.5767; post-event *r* = 0.6874; Δ *r* = 0.4803). Internal consistency measured by Cronbach’s alpha were of 0.9302 for Hardiness values, 0.9442 for Emotional Intelligence and 0.9568 for the two instruments assessed together.

**TABLE 2:** Hardiness and EQ score means and standard deviations of pre-event, post-event, and difference (Δ) (post-event – pre-event scores).

**Variable**	**Pre**		**Post**		**Difference (Δ)**	
	**Mean**	**Std. Dev.**	**Mean**	**Std. Dev.**	**Mean**	**Std. Dev.**
**Hardiness**	111.63	8.95	116.31	9.89	5.34	8.55
Control	111.19	10.97	115.63	12.19	4.68	5.86
Commit	108.66	11.95	114.06	11.82	4.44	8.04
Challenge	112.40	10.79	117.74	10.69	5.40	7.59
**EQ**	104.57	10.76	108.65	12.21	4.07	6.97
SP	104.50	10.54	108.53	10.83	4.03	6.88
SE	102.03	13.07	104.03	14.07	2.00	7.82
IS	103.94	10.83	107.69	9.87	3.75	6.95
DM	104.26	11.29	106.85	12.81	2.59	9.61
SM	104.21	13.68	108.76	14.41	4.56	8.21

*EQ, Emotional Quotient, SP, Self Perception, SE, Self Expression, IS, Interpersonal, DM, Decision Making, SM, Stress Management. Sub scores for each instrument are also described.*

**FIGURE 1:**
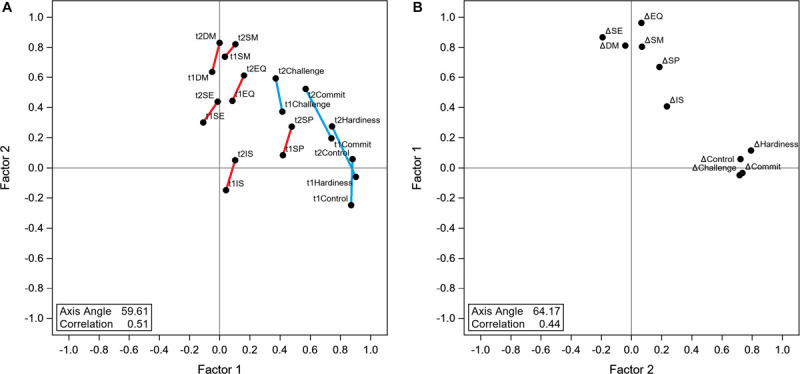
Factor analysis loading scores of Hardiness and Emotional quotient (EQ). **(A)** Pre-event (t1) and Post-event (t2) loadings plot. Panel shows an Optimin rotation of factor loadings. Pre and post data point are linked by a line to help visualize their spread. Hardiness data points are linked in blue while Emotional Intelligence are linked in red. **(B)** Difference (Δ) loadings plot. Panel shows an Optimin rotation of factor loadings and has axes flipped to match Panel **(A)** loading orientation.

Score variation patterns are displayed for the first two dimensions (factors) although all retained factors were included in the Oblimin rotation. These patterns are displayed in Figure 1. Pre-event and post-event total variance explained was 73.27% for the three factors retained, while Δ total variance explained was 61.48% for two factors retained. For the pre-event and post-event overall pattern (Figure 1A), the Oblimin rotation aligned very closely each of the two data types Hardiness and Emotional Intelligence into each of the perpendicular axes. Although some specific sub scores displayed overlap, most scores and sub scores by data type aligned to their axes. This pattern suggests some sub scores may be capturing a similar trait, the most obvious overlaps were observed from Challenge and Commit sub scores for HRG and for Self-Perception and Interpersonal for EQ-I2.0; these place between the axes. In addition, the largest shift from Pre to Post was observed in Hardiness HRG total scores and sub scores. For Δ scores and sub scores (Figure 1B), the pattern was very similar with less overlap. Hardiness Δ scores and sub scores cluster closely along their axis while Emotional Intelligence Δ scores and sub scores cluster similarly with a slight difference on the perperdicular axis.

Mixed model assessment of age, sex and time point along with the sex by time point interaction effects are presented in Table 3. A very consistent effect of time point was observed where the effect was Bonferroni significant for all but two Emotional Intelligence sub scores (Self Expression and Decision Making). For Hardiness, the largest significant increase was for the Challenge sub score (6.38 ± 1.18 points) and the smallest significant increase was for the Control sub score (5.06 ± 1.12 points). For Emotional Intelligence, the largest significant increase was for the Stress Management sub score (5.41 ± 1.14) and the smallest significant increase was for the Self Expression sub score (2.53 ± 1.10). This pattern of statistical significance strongly suggest that a 6-day long training session does improve Hardiness and Emotional Intelligence scores. No sex effects were observed except for a single instance when evaluating the sex by time point interaction (Hardiness main score); however, after further examination, none of the Male Vs. Female pairwise comparisons at a specific given time point were significant (“Sex| Time point = Pre”, *P* = 0.9236; “Sex | Time point = post” *P* = 0.1908). This suggests men and women benefit equally from the training session. Age does have an effect on scores and sub scores where older age is associated with higher scores. For Hardiness, the Challenge sub score was the only factor that was significantly associated to age (0.88 ± 0.35 point increase per year of age). For Emotional Intelligence, the largest significant increase was for the Self Expression sub score (1.07 ± 0.46 point increase per year of age) and the smallest significant increase was for the Decision Making sub score (0.86 ± 0.40 point increase per year of age); in addition, the EQ main score displayed a significant increase associated to age (0.95 ± 0.39 point increase per year of age). This effect indicates that older age is associated to better total scores and sub scores but only on specific components.

**TABLE 3:** Effect of age, sex, location, and sex by time point interaction on Hardiness and EQ scores and sub scores.

	**Effect**			
	**Age *P***	**Sex *P***	**Time point *P***	**Sex by time point *P***
**Hardiness**	0.1533	0.4596	3.10E-09**	0.0466*
Control	0.1324	0.7885	2.81E-05**	0.2757
Commit	0.1405	0.0611	1.18E-06**	0.5529
Challenge	0.0134*	0.0601	9.11E-07**	0.0809
**EQ**	0.0172*	0.0752	4.43E-06**	0.1205
SP	0.1443	0.1370	1.90E-05**	0.3857
SE	0.0238*	0.0903	0.0242*	0.3376
IS	0.1915	0.0885	1.09E-04**	0.5699
DM	0.0344*	0.1894	0.0075*	0.1095
SM	0.0448*	0.3492	1.13E-05**	0.1390

*Significant at *P* ≤ 0.05 confidence threshold but not at a family-wise Bonferroni adjusted confidence threshold.**Significant at a family-wise Bonferroni adjusted confidence threshold.

Mixed model assessment of age, sex on Difference (Δ) scores and sub scores showed a different picture, these values are presented in Table 4. Just as in the pre-event vs. post-event models, sex was not statistically significant in any instance. Age was only significant for the Self Perception sub score of Emotional Intelligence where being younger provided an advantage for a higher improvement (0.52 ± 0.25 point decrease per year of age).

**TABLE 4:** Effect of age and sex on difference (Δ) Hardiness and EQ scores and sub scores.

	**Effect**	
	**Age *P***	**Sex *P***
**Δ Hardiness**	0.7434	0.0517
Δ Control	0.9823	0.2864
Δ Commit	0.3983	0.4764
Δ Challenge	0.6575	0.0988
**Δ EQ**	0.2298	0.1729
Δ SP	0.0404*	0.5703
Δ SE	0.4127	0.4104
Δ IS	0.9346	0.5856
Δ DM	0.2396	0.1503
Δ SM	0.4749	0.1692

*Significant at *P* ≤ 0.05 confidence threshold but not at a family-wise Bonferroni adjusted confidence threshold.

In summary, our results show that Hardiness and Emotional Intelligence total scores and sub scores are improved by participating in the hyper realistic training session. The improvement is not associated to sex and is only associated to age for particular components. In addition, we observed that Hardiness measured through the HRG instrument and Emotional Intelligence measured thorough the EQ-I2.0 instrument measure predominantly different traits even when there is a slight overlap that may be captured by both.

## Discussion

There is strong evidence supporting the use of Hardiness and Emotional Intelligence’s for the development of strategies to mitigate risks of developing detrimental burnout and post-traumatic stress symptoms (Escolas et al., 2013). Interventions to improve Hardiness and Emotional Intelligence have shown positive impact reducing depression symptoms in college students (Kuk et al., 2019); and reducing depression, anxiety and physical complaints of college students (Kalantar et al., 2013). Altough no evaluations have been performed that can establish any definitive long term effects of a short intervention, a single study shows some promise, where a 2-h Emotional Intelligence training session given to Emergency Medicine residents, an increase in scores is still detectable 6 months after the initial intervention (Gorgas et al., 2015). Long term monitoring of participants would be necessary to establish long term benefits. The positive impact of Hyper Realistic Simulation Training^®^ over team performance of US military active personel has been documented (Hoang et al., 2016). It was hypothesized that this training would have a positive effect on improving both Hardiness and Emotional Intelligence and that improvement would appear in specific sub components that guide the design for improved training programs. The present study adds to the list of positive reports and provides additional comparisons where two separate potentially overlapping concepts are evaluated simultaneously. The data illustrated that a 6-day hyper-realistic simulation improved both Hardiness and Emotional Intelligence of medical personnel. Our findings support what was proposed by LaPorta and collaborators in 2017; hyper-realistic teaching methodologies improve trainee effectiveness (LaPorta et al., 2017).

Although the definition of Hardiness and Emotional Intelligence concepts are well-defined theoretical concepts, the nuances are difficult to capture on an instrument. These traits are derivatives of complex neurological and physiological processes. There is an association of Emotional Intelligence and brain network topology (Ling et al., 2019). These biological systems are often intimately interconnected with each other. For that reason, it is reasonable to believe there is an overlap in Hardiness and Emotional Intelligence. Unfortunately, no previous reports comparing these two instruments side to side were available. Significant correlations between the two instruments suggest that an individual’s score for one instrument broadly predicts the other score. However, this can be an over simplified interpretation of the overlap. Factor analysis of total scores and sub scores provided a more detailed description of the instruments overlap. After visualization rotation, a clear overall alignment on separate axes for each instrument was observed with some specific sub scores appearing in between both axes. These sub scores appearing in between the axes are indicative of overlap (Challenge and Commitment from HRG and Self Perception and Interpersonal from EQ-I2.0). It is unclear how these elements interact with each other; however, based on their descriptions we can speculate that they describe similar concepts: on one side within the HRG, Challenge describes the perception of the situation in terms of being a challenge or opportunity while Commitment evaluated the self-perceived importance of their work; on the other side within the EQ-I2.0, Self-Perception evaluates perceptions of self-regard, self-actualization, and emotional self-awareness while Interpersonal determines the value of interpersonal relationships, empathy and social responsibility. The concepts included in these definitions overlap in their semantics and practical situational applications and therefore require clever design of stimulus to separate their effects. This phenomenon is not unexpected and is likely to occur in a similar pattern with other assessment instrument not included in this study (Galaiya et al., 2020). The way the instruments are structured is crucial for their interpretation even when they are aimed toward similar or even the same goals.

Sex and age differences in Emotional Intelligence have been evaluated in the past in several contexts (Arora et al., 2010; Davis and Nichols, 2016); however, the modeling approach used in this study evaluates the effect of sex an age in the context of its relevance in an intervention. The results presented do not provide evidence of differential sex benefit but do so for age. If sex differences would exist, the effect size is likely to be very small which would require a much larger sample to be detected. This outcome may be inherently distorted by the participant type evaluated in the study. All participants in the present study are military medical students which may diverge from generalized medical personel trends. The specific performance requirements along with associated restrictions for this career path may help explain what was observed and should be investigated in future studies. These findings are important for the development of efficient interventions for similar populations or populations in similar occupational fields.

The main limitations in this study are associated to its intervention setting along with its highly specialized population. These two factors can complicate the interpretation of the results to infer on more generalized populations. Although a hyper-realistic immersive surgical training may be most applicable to surgery and emergency medicine, but the purpose of Hardiness and Emotional Intelligence is applicable to all specialities. In addition, due to the military restrictions on eligible participants, no feasible control groups can be introduced to witness the effect of the intervention in the same 6-day time frame. This limitation could be bypassed by following the development of the participants of this study and or of similar ones to determine the long-term effects of these types of interventions when compared to personnel not exposed to Hardiness and Emotional Intelligence training inside and outside of military service. To this moment, some of the elements employed in the hyper realistic approach are already being used to train medical personel in the civilian sector, such as the use of “cut suits” for training first responders to address hemorrhage control in austere environments (Kirkpatrick et al., 2017). The transfer of the technology and interventions used in this study, that were developed originaly for military training, is expected to expand in the near future as civilian organizations in charge of managing emergency respose services along with medical professionals in emergency medicine fields recognize the benefits of such training. For that reason it is important to evaluate and carefuly define the repercusions of these types of approach. An important question related to hyper-realistic immersion training is its accuracy toward the real event reenacted and the potential to cause unintended psychological distress. Even when the event is a simulation, there is a large effort to make the event as realistic as possible and therefore the intensity of the stimulus provided could be high enough to cause emotional harm to participants. This concern can also be addressed by a debrief of particiapants for long term effects. An evaluation of long term beneficial or detrimental effects of this type of intervention is a priority and is currently an ongoing project.

## Conclusion

In summary, a 6-day hyper-realistic immersive surgical training for military medical students increased Hardiness and Emotional Intelligence scores. The improvement observed does not disproportionately benefit one sex over the other. However, older participants have significantly higher values for Emotional Intelligence total score along with the Challenge, Self-Expression, Decision Making, and Stress management sub scores. Additionaly, older participants had improvement in their Self Perception sub score. Finally, a simultaneous evaluation of each instrument showed predominant differences in capturing individual’s traits with some overlap in specific scores. Results from this study support the implementation of hyper realistic interventions to increase Hardiness and Emotional Intelligence in medical trainees for mitigation of burnout and posttraumatic stress symptoms.

## Data Availability Statement

De-identified raw data supporting the conclusions of this article will be made available by the authors, without undue reservation.

## Ethics Statement

The studies involving human participants were reviewed and approved by IRB Rocky Vista University (RVU IRB 2014-0001). The patients/participants provided their written informed consent to participate in this study.

## Author Contributions

AW coordinated the training session and drafter the manuscript. IZ and RR performed the analysis and drafted the manuscript. AL assisted in the data management and drafted the manuscript. NN, TH, RF, MS, DC, and AL envisioned, designed, and coordinated the training session. All authors contributed to the article and approved the submitted version.

## Conflict of Interest

The authors declare that the research was conducted in the absence of any commercial or financial relationships that could be construed as a potential conflict of interest.
